# Relationship between insecticide resistance and *kdr* mutations in the dengue vector *Aedes aegypti* in Southern China

**DOI:** 10.1186/s13071-015-0933-z

**Published:** 2015-06-12

**Authors:** Chun-Xiao Li, Phillip E Kaufman, Rui-De Xue, Ming-Hui Zhao, Gang Wang, Ting Yan, Xiao-Xia Guo, Ying-Mei Zhang, Yan-De Dong, Dan Xing, Heng-Duan Zhang, Tong-Yan Zhao

**Affiliations:** Department of Vector Biology and Control, State Key Laboratory of Pathogen and Biosecurity, Institute of Microbiology and Epidemiology, Beijing, 100071 China; Entomology & Nematology Department, University of Florida, Gainesville, FL 32611 USA; Anastasia Mosquito Control District, 500 Old Beach Road, St. Augustine, FL USA

**Keywords:** *Aedes aegypti*, *kdr* mutation, China

## Abstract

**Background:**

*Aedes aegypti* is an important vector for dengue virus and thus has been targeted with pyrethroid insecticides in many areas of the world. As such, resistance has been detected to several of these insecticides, including in China, but the mechanisms of the resistance are not well understood in this country.

**Methods:**

Using the World Health Organization larval mosquito bioassay, five field populations of *Aedes aegypti* from Southern China were characterized for their resistance to cypermethrin and cyhalothrin. RNA extraction with PCR amplification, cloning and sequencing of the sodium channel gene was followed by comparisons of susceptible and wild mosquito strains Additionally, genomic DNA was used for Allele-specific PCR (AS-PCR) genotyping of the sodium channel genes to detect S989P, V1016G and F1534C mutations and allow for correlation analysis of resistance expression for the different mutations.

**Results:**

All wild strains expressed resistance to cypermethrin and cyhalothrin and the resistance expression between the two insecticides was highly correlated suggesting cross-resistance between these two pyrethroids. The AS-PCR technique effectively distinguished individual genotypes for all three mutations. Among the five wild strains tested, two strains carried all three mutations. Although the S989P and V1016G mutations were positively correlated to resistance expression of both pyrethroids, the F1534C mutation was negatively correlated.

**Conclusions:**

Our methodology proved highly reliable and will aid future detection of *kdr* mutations. The three sodium channel mutations were common in the *Ae. aegypti* strains sampled from Southern China. The V1016G mutation appears to be the most important *kdr* mutation in *Ae. aegypti* strains in Southern China.

## Background

The mosquito *Aedes aegypti* (L.), a primary vector of dengue fever and dengue hemorrhagic fever, mainly occurs in tropical areas. Because there is currently no dengue vaccine, mosquito control has become the most important method of preventing transmission of these diseases [1] and insecticides are expected to remain a key component of dengue control for the foreseeable future [2]. Pyrethroids, characterized by their high toxicity to target insects and relatively few adverse effects on mammals, have been used widely in mosquito control. However, many mosquito populations have developed resistance to these compounds following repeated exposure to them [3, 4].

Both pyrethroids and DDT target the voltage-gated sodium channel of insect neurons and single amino acid substitutions in the sodium channel have been associated with resistance to both these insecticides [5]. This form of resistance, known as knockdown resistance (*kdr*), has been observed in several insect species including the mosquitoes *Anopheles gambiae* Giles [6], *An. stephensi* Liston [7], *Culex quinquefasciatus* Say [8] and *Ae. aegypti* [9].

In many insect species, mutations related to both pyrethroid and DDT resistance largely have been located in the IIS6 region. Of these, the replacement of a Leu for a Phe in the 1014 site (Leu1014Phe), first described in a DDT resistant strain of *Musca domestica* L. [10], was the most common. Other substitutions in the same homologous site have been described in a number of species, such as Leu1014Ser in the mosquitoes *An. gambiae* [11] and *Culex pipiens* L. [12] and Leu1014His in the tobacco budworm *Heliothis virescens* (Fabricius) [13]. In *Ae. aegypti*, substitutions at site 1014 are unlikely, as two independent changes in the same codon would be necessary [14]. Instead, mutations in different positions have been observed in Latin American and Southeast Asian populations of *Ae. aegypti*. So far, a number of *kdr* mutations have been identified in *Ae. aegypti* (S989P, I1011M/V, V1016G/I, F1269C, F1534C) [9, 14, 15] and at least two of these are known to be related to pyrethroid resistance; 1016 (Val to Ile or Gly) and 1534 (Phe to Cys) in the IIS6 and IIIS6 segments of the Vssc gene, respectively [9, 14, 16, 17].

*Ae. aegypti* is present in many residential areas of Southern China. In recent years, regular monitoring has revealed that many Chinese populations of *Ae. aegypti* have become resistant to pyrethroid insecticides [18, 19], but the underlying mechanism responsible for this resistance is unknown. The aims of this study were to detect and identify mutations in the sodium channel gene and to determine whether any of these are linked to pyrethroid resistance in wild *Ae. aegypti* populations in China. We used the WHO susceptibility test to evaluate the resistance of *Ae. aegypti* larvae to commonly used pyrethroid insecticides and molecular assays to identify specific point mutations in the sodium channel gene of *Ae. aegypti* collected from various regions in Southern China. The relationship between pyrethroid knockdown resistance and the frequencies of different *kdr* alleles is analysed and discussed. A novel method has also been developed to detect the mutation found in Chinese *Ae. aegypti* field strains and to determine the role of the mutations in cypermethrin and cyhalothrin resistance in China. Determining the distribution of one of the major mechanisms underpinning *kdr* mutations in wild mosquito populations is important to improving the effectiveness of dengue control operations.

## Methods

### Mosquito specimens

Larvae of *Ae. aegypti* were collected from five sites in Southern China in 2013: Qishui (20°45′46.3″N, 109°45′35.0″E), Dongfang (19°6′3.8″N, 108°35′8.6″E), Jinghan (24°01′51.5″N, 97°53′15.6″E), Yundang (24°01′25.6″N, 97°52′58.9″E) and Munao (24°01′08.6″N, 97°51′47.8″E). Larvae were collected from standing water sources such as jars, bowls, tanks, tin cans and drums. No specific permissions were required to collect larvae as their collection did not involve, or affect, any endangered or protected species. Collected larvae were transported to the laboratory and colonized. Bioassays for pesticide resistance were conducted on the first (F_1_) generation of larvae produced in captivity and the results compared to those of a pyrethroid-susceptible *Ae. aegypti* strain. The susceptible *Ae. aegypti* strain originated from Hainan province, China, and has been isolated in a laboratory for more than 10 years without exposure to insecticides. Larvae were flash-frozen at −70 °C after pesticide exposure trials.

### Mosquito bioassay

The resistance of larvae to two pyrethroids, cypermethrin (≥98 %, Sigma, USA) and cyhalothrin (>99 %, Sigma, USA), was evaluated. Thirty late 3rd and early 4th instar larvae were placed in a glass container that held 199 ml water and 1 mL of an insecticide concentration using the methods outlined by the WHO [20]. All analytical grade insecticides were diluted five to seven concentrations with acetone. The serial concentrations were 0.0002, 0.0005, 0.001, 0.003, 0.005, 0.008 and 0.01 ppm for the susceptible strain, Qishui strain and Dongfang strain. The serial concentrations were 0.005, 0.01, 0.03, 0.05, 0.08 ppm for Jinghan strain and Yundang strain. The serial concentrations were 0.025, 0.05, 0.1, 0.15 and 0.2 ppm for Munao strain. A control with no insecticide was done in order to control for the natural mortality of the strain tested. All experiments were repeated three times and larval mortality recorded 24 h after treatment. Larval mortality was determined by dividing the number of dead larvae by the total number tested. No food was provided to larvae during bioassays, which were conducted under a 14 L: 10D photoperiod, 75 % relative humidity and temperature of 26 ± 1 °C.

LC_50_ values were calculated using Schoofs and Willhite’s [21] probit analysis program. As a measure of resistance we calculated the resistance ratio (RR) [22], which was the ratio of the estimated LC_50_ of the F_1_ captive generation to that of larvae of the susceptible strain. In order to examine whether resistance was correlated among sampling sites, relationships between estimated RR values of various insecticides were analyzed by Pearson correlation analysis. *P*-values <0.05 were considered statistically significant.

### Extraction of mosquito RNA and synthesis of mosquito cDNA

Total RNA of 30 female mosquitoes from the susceptible laboratory strain and the five wild strains were extracted with TRIzol reagent according to the manufacturer’s protocol (Invitrogen, U.S.A.). These samples came from the same generation and age classes (late 3rd and early 4th instar) used for the resistance bioassay. The yield and purity of the extracted RNA were assessed by determining its absorbance (Abs) at 260 and 280 nm. RNA was used only if its Abs260 nm/Abs280 nm ratio was >1.8. Extracted total RNA was stored at −70 °C for later use.

Two microliters of total RNA from each sample was used to synthesize the first-strand cDNA using a oligo(dT) _12–18_ primer in a 20-μl reaction following the Superscript ^TM^ RT-PCR System (Invitrogen, U.S.A.). The first-strand cDNA reaction was stored at −20 °C until required.

### PCR amplification of the *Ae. aegypti* sodium channel gene

Gene-specific primers based on the published sequence of the *Ae. aegypti* para-sodium channel gene (GenBank Accession No.: EU399179.1) were designed in NCBI Primer BLAST and used to amplify the sodium channel gene of specimens from each strain. The sodium channel gene is 6554 bp in length and was amplified as 11 fragments. The primers used are shown in Table 1. The PCR reaction consisted of one cycle of 94 °C for 3 min and 35 cycles at 94 °C for 30 s, 46 ~ 63 °C for 30 s (different Tm for 11 fragments’ amplification), 72 °C for 1 min and a final extension step at 72 °C for 7 min.

**Table 1:** The eleven pairs of primers used to amplify the *Ae. aegypti* sodium channel gene

Section	Primer (5′-3′)	Beginning nucleotide	End nucleotide	Length (bp)	Tm
I	GACAATGACCGAAGACTC	17	693	677	46 °C
	ATGCTAATGCTATTACTACG				
II	GCGAGGTTTCATATTACAA	614	1262	649	49 °C
	ACCCAAGAAGATAATCACAA				
III	CGTGGCACATGCTCTTCT	1222	1833	612	55 °C
	TGACCGCGTTCGAGTCAT				
IV	CACAAGAACATTTGCCGTAC	1792	2401	610	53 °C
	TCCTGGAACTTGAGCCAC				
V	GGCTCAAGTTCCAGGAGT	2386	3012	627	51 °C
	CACCCACAAGCATACAATC				
VI	GTGGGATTGTATGCTTGTG	2990	3592	603	52 °C
	ATGCCTCTATGATTCAGTTCGT				
VII	CCAAGGTGATAGGCAATT	3520	4156	637	51 °C
	CAGGCGTTCGTAAAGTAAA				
VIII	CAAGTGGTTGGCGCTGGGTT	4112	4768	657	63 °C
	CCGGCTTTCTTCTTCTGTTCGT				
IX	TTCATCATCTTCGGGTCG	4683	5326	644	52 °C
	AAGAACAGCAGCAGACAGA				
X	GTCAAGGGTGCCAAAGGT	5238	5914	677	55 °C
	TTCCGAGCGAAGAAGTCC				
XI	ACATACCGATCTGTCGAG	5830	6495	660	47 °C
	ATTTCTGTCGTGCTTCTG				

### Cloning and sequencing of PCR products

PCR products were run on a 1.5 % agarose gel and purified using Wizard PCR Preps DNA Purification System (Promega, U.S.A.). Purified PCR products were ligated into pMD 18-T easy vector (Takara, Japan) and plasmids cloned into *E. coli* XL1-blue competent cells. Plasmids were purified using a miniBEST plasmid purification kit (Takara, Japan). At least 30 clones of each PCR product were selected. Sequencing reactions were carried out on recombinant plasmids using M13 forward and reverse primers. Sequence alignment was performed with the Clustal W algorithm [23].

### Comparison of sodium channel genes from susceptible and wild strains

The amino acid and cDNA sequences of susceptible and wild strains collected in 2013 were analysed with DNAstar software [24]. Blast research was performed at http://www.ncbi.nlm.nih.gov/blast/. Sequences were aligned with ClustalX software [23] and viewed with GeneDoc software [25].

### Genomic DNA isolation

Genomic DNA was isolated from 32 ~ 36 female mosquitoes from each strain using a Universal Genomic DNA Extraction Kit according to the manufacturer’s protocol (Takara, Japan). DNA was solubilized in a final volume of 100 μl ddH_2_O.

### Allele-specific PCR (AS-PCR) genotyping of sodium channel genes

**Fig. 1:**
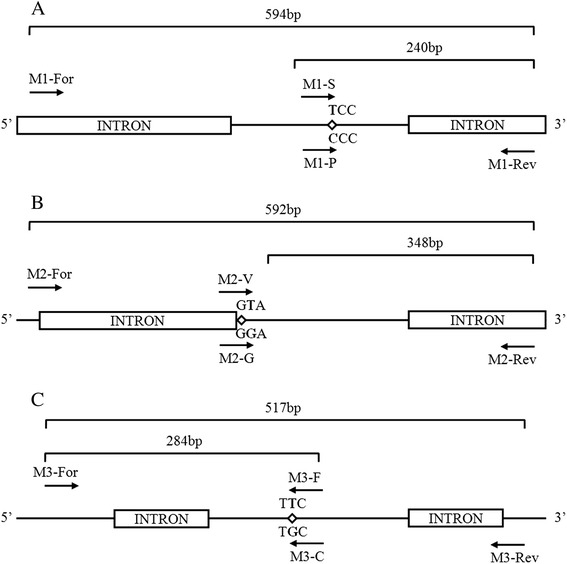
Schematic diagram of AS-PCR test used to detect the S989P (**a**), V1016G (**b**) and F1534C (**c**) mutations in the sodium channel gene of *Ae. aegypti*

AS-PCR was used to detect S989P, V1016G and F1534C mutations in each of the five wild *Ae. aegypti* strains. A test using two PCR reactions for each individual was developed to diagnose each mutation (Fig. 1). The two reactions were exactly the same except that one contained a susceptible-specific primer and the other contained a mutant-specific primer. The outer primers and allele-specific inner primers for the three target mutations are listed in Table 2. A PCR diagnostic test was performed in accordance with the standard procedure with a total reaction volume of 25 μl, consisting of 80–100 ng genomic DNA, 14 mM Tris–HCl, pH 8.3, 70 mM KCl, 4.5 mM MgCl2, 0.15 mM each dNTP and 0.67 U TakaRa rTaq. PCR conditions were one cycle of 94 °C for 3 min, then 35 cycles of 94 °C for 30s, 60 or 62 °C (60 °C for V1016G and F1534C, 62 °C for S989P) for 30 s and 72 °C for 1 min, followed by one cycle of 72 °C for 7 min. PCR products were checked by electrophoresis on 1.5 % agarose gel in TAE buffer. Bands were visualized by ethidium bromide staining. The size of the PCR products for the detection of *kdr* alleles were 240 bp (S989P), 348 bp (V1016G) and 284 bp (F1534C), whereas the size of the products used as allele-nonspecific outer primers were 594 bp (S989P), 592 bp (V1016G) and 517 bp (F1534C).

**Table 2:** The specific primers used to amplify sodium channel gene mutations detected in *Ae. aegypti*

Mutation	Primers	Sequence (5′-3′)
S989P	M1-For	AATGATATTAACAAAATTGCGC
	M2-Rev	GCACGCCTCTAATATTGATGC
	M1-S	GCGGCGAGTGGATCGAAT
	M1-P	GCGGCGAGTGGATCGAAC
V1016G	M2-For	GCCACCGTAGTGATAGGAAATC
	M2-Rev	CGGGTTAAGTTTCGTTTAGTAGC
	M2-V	GTTTCCCACTCGCACAGGT
	M2-G	GTTTCCCACTCGCACAGGG
F1534C	M3-For	GGAGAACTACACGTGGGAGAAC
	M3-Rev	CGCCACTGAAATTGAGAATAGC
	M3-F	GCGTGAAGAACGACCCGA
	M3-C	GCGTGAAGAACGACCCGC

### Correlation of resistance with the frequencies of different mutations

In order to assess whether the frequency of different *kdr* alleles in *Ae. aegypti* strains was related to resistance, the relationship between allele frequency and the LC_50_ values for cypermethrin and cyhalothrin was analysed with SAS Software 6.22 (SAS Institute Inc.). Coefficients of correlation were calculated between the frequency of different mutations in different strains and the corresponding LC_50_ values for each pyrethroid. To determine whether the correlation was significant, the null hypothesis of r = 0, *i.e.* no correlation, was tested with a Student’s *t*-test. Significance levels of these tests were adjusted using the Bonferroni Correction [26] to compensate for the increased likelihood of obtaining significant results by chance.

## Results

### Correlation of resistance to different insecticides

**Fig. 2:**
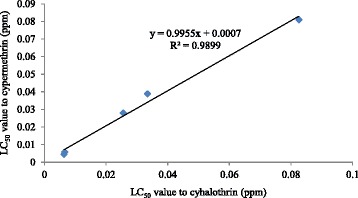
Relationship between LC_50_ to cypermethrin and cyhalothrin in five wild strains of *Ae. aegypti*

All wild strains had various levels of resistance to the insecticides tested (Table 3). The resistance ratio (RR) of each strain for cypermethrin and cyhalothrin ranged from 6.82 to 88.82-fold and 4.82 to 88.87-fold, respectively. The Munao strain had the highest resistance to these two pyrethroids. Resistance to cypermethrin and cyhalothrin was significantly correlated, *i.e.* strains with high resistance to cypermethrin tended to also be highly resistant to cyhalothrin (R^2^ > 0.9, *P* < 0.05) (Fig. 2) which indicates cross-resistance to these two insecticides.

**Table 3:** Levels of cypermethrin and cyhalothrin resistance in different strains of *Ae. aegypti*

Insecticide	Strain	LC_50_ (ppm) (95 % CL)	RR^a^	Regression equations
Cypermethrin	SS	0.00093 (0.00086–0.00100)	1	Y = 12.04407 + 3.97312X
	QS	0.00663 (0.00628–0.00701)	7.13	Y = 13.24042 + 6.07821X
	DF	0.00634 (0.00490–0.00927)	6.82	Y = 5.75003 + 2.61641X
	JH	0.03349 (0.02218–0.06264)	36.01	Y = 2.77811 + 1.88336X
	YD	0.02558 (0.02303–0.02834)	27.51	Y = 3.86610 + 2.42842X
	MN	0.08260 (0.07227–0.09338)	88.82	Y = 2.45754 + 2.26918X
Cyhalothrin	SS	0.00091 (0.00079–0.00105)	1	Y = 7.75649 + 2.55102X
	QS	0.00568 (0.00531–0.00607)	6.24	Y = 9.59773 + 4.27341X
	DF	0.00439 (0.00382–0.00505)	4.82	Y = 7.39856 + 3.13863X
	JH	0.03879 (0.03518–0.04299)	42.63	Y = 4.90089 + 3.47272X
	YD	0.02794 (0.02530–0.03092)	30.70	Y = 3.79551 + 2.44265X
	MN	0.08087 (0.06909–0.09386)	88.87	Y = 2.59628 + 2.37709X

*SS* susceptible strain, *QS* Qishui strain, *DF* Dongfang strain, *JH* Jinghan strain, *YD* Yundang strain, *MN* Munao strain

^a^RR = resistance ratio. When the non-insecticide control was used, the percentage of natural mortality is 2.2 %, 1.1 %, 0 %, 1.1 %, 0 % and 0 % for SS, QS, DF, JH, YD and MN strain

### Identification of sodium channel gene mutations

Three mutations were identified from the cDNA of a pooled sample of F_1_ specimens of wild *Ae. aegypti*: S989P (TCC-CCC), V1016G (GTA-GGA), and F1534C (TTC-TGC), compared to the susceptible strain.

### AS-PCR genotyping of mosquitoes carrying *kdr* mutations

**Fig. 3:**
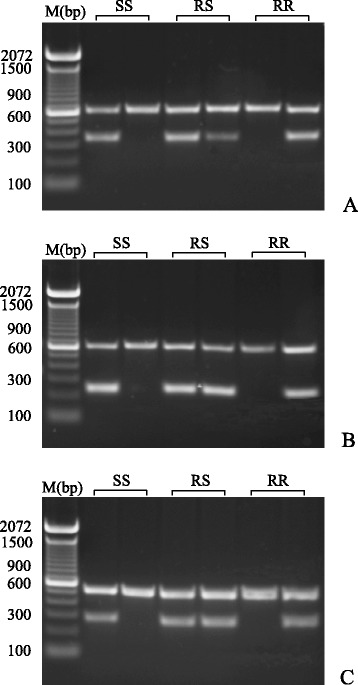
Gel electrophoresis bands of AS-PCR products corresponding to the *Ae. aegypti* sodium channel gene mutations S989P (**a**), V1016G (**b**) and F1534C (**c**). M: marker

After optimizing experimental conditions, AS-PCR could effectively distinguish individual mosquitoes that were homozygous or heterozygous for the S989P, V1016G and F1534C mutations (Fig. 3). All five wild strains were tested and the mutation frequencies are summarized in Table 4. The Qishui and Dongfang strains had no mutations at sites S989 and V1016, however, three strains from Ruili (Jinghan, Yundang and Munao) had high frequencies of these mutations which reached 100 % in the Munao strain. At the F1534 locus, the opposite situation was apparent; mutation frequency was 100 % in the Qishui strain but very low in the three Ruili strains, indeed, there were no mutations at this locus in the Munao strain. The Jinghan and Yundang strains carried all three mutations.

**Table 4:** Frequency of the S989P, V1016G and F1534C mutations in the sodium channel gene of five wild strains of *Ae. aegypti*

Mutation	Strain	Genotype frequency (%)			
		SS	RS	RR	R
S989P	QS	100.0	0.0	0.0	0.0
	DF	100.0	0.0	0.0	0.0
	JH	2.8	30.6	66.7	81.9
	YD	0.0	33.3	66.7	83.3
	MN	0.0	0.0	100.0	100.0
V1016G	QS	100.0	0.0	0.0	0.0
	DF	100.0	0.0	0.0	0.0
	JH	0.0	16.7	83.3	91.7
	YD	0.0	9.1	90.9	95.5
	MN	0.0	0.0	100.0	100.0
F1534C	QS	0.0	0.0	100.0	100.0
	DF	3.0	18.2	78.8	87.9
	JH	84.8	15.2	0.0	7.6
	YD	90.9	9.1	0.0	4.6
	MN	100.0	0.0	0.0	0.0

SS: susceptible homozygote. SS% = SS/(SS + RS + RR) × 100 %

RS: mutant heterozygote. RS% = RS/(SS + RS + RR) × 100 %

RR: mutant homozygote. RR% = RR/(SS + RS + RR) × 100 %

R% = RR% + 0.5 × RS%

*QS* Qishui strain, *DF* Dongfang strain, *JH* Jinghan strain, *YD* Yundang strain, *MN* Munao strain

### Correlation between insecticide resistance and mutation frequency

**Fig. 4:**
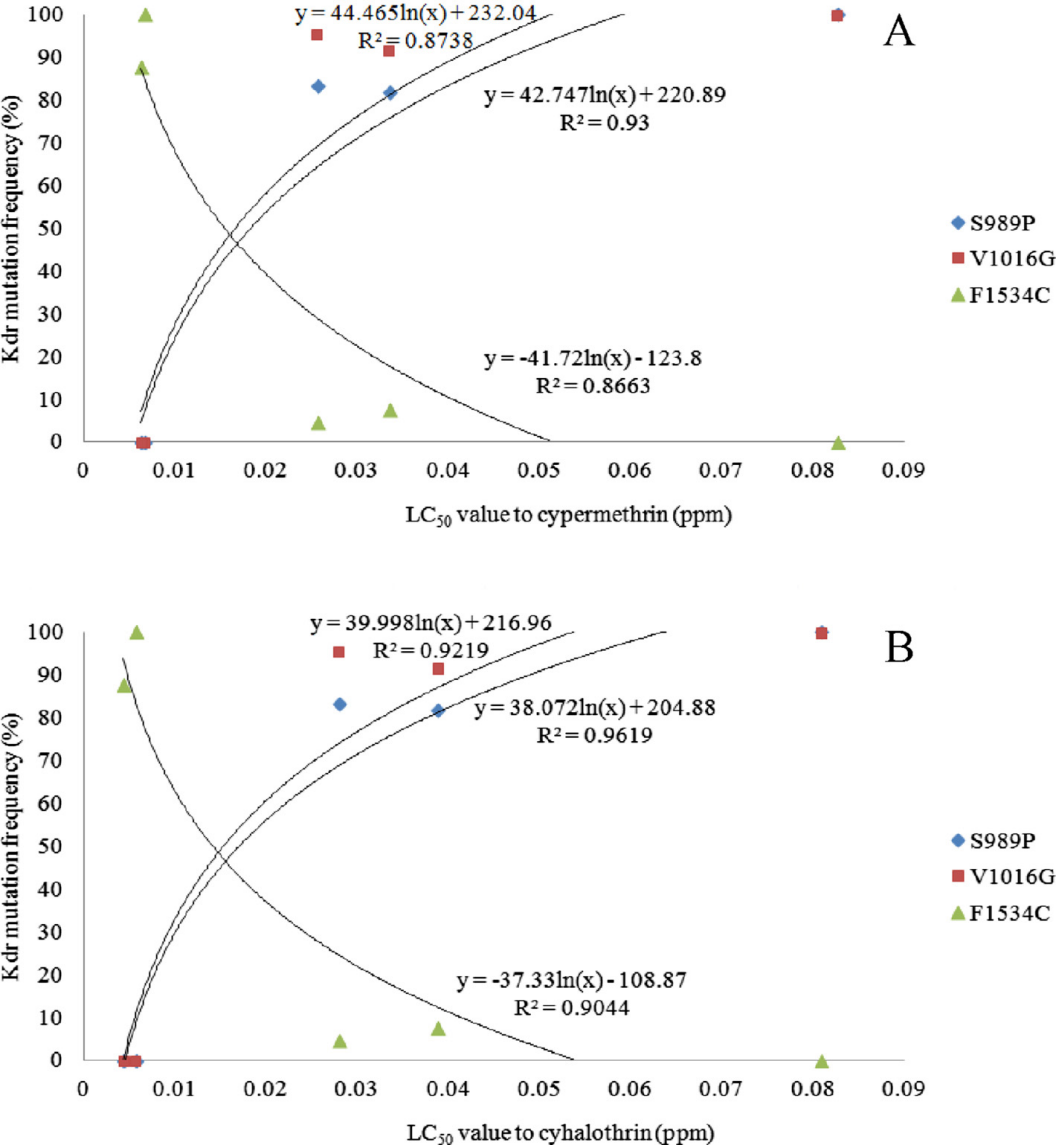
Relationship between frequency of the S989P (◆), V1016G (■) and F1534C (▲) *kdr* mutations and LC_50_ to cypermethrin (**a**) and cyhalothrin (**b**)

A significant positive correlation between the frequency of the S989P and V1016G point mutations and resistance to cypermethrin and cyhalothrin was observed when comparing LC_50_ values and the frequency of these sodium channel gene mutations (Fig. 4). However, the frequency of the F1534C point mutation was negatively correlated with resistance to these two pyrethroids (Fig. 4).

### Correlation between the S989P and V1016G mutations

**Fig. 5:**
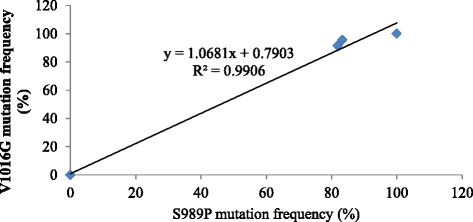
Correlation between the frequency of the S989P and V1016G mutations

The results show a strong linear relationship between the frequency of the S989P and V1016G mutations (Fig. 5). Co-occurrence of the S989P and V1016G mutations in five wild strains are presented (Table 5)*.* The Qishui and Dongfang strains were all SS homozygotes and the Munao strain were all RR homozygotes, but the Jinghan and Yundang strains were either RS heterozygotes (13.9 % and 9.1 %) or RR homozygotes (66.7 %). In the remaining small proportion of individual mosquitoes, if the S989P mutation was detected as SS, the V1016G mutation was expressed as RS (2.8 % in Jinghan strain). Whereas, if the S989P mutation was expressed as RS, the V1016G mutation was expressed as RR (16.7 % in Jinghan strain, 24.2 % in Yundang strain).

**Table 5:** Co-occurrence of the S989P and V1016G mutations in five wild strains of *Ae. aegypti*

Mutation	Strain	Genotype frequency (%)								
		S/S989 + V/V1016	S/P989 + V/V1016	P/P989 + V/V1016	S/S989 + V/G1016	S/P989 + V/G1016	P/P989 + V/G1016	S/S989 + G/G1016	S/P989 + G/G1016	P/P989 + G/G1016
S989P + V1016G	QS	100.0	0.0	0.0	0.0	0.0	0.0	0.0	0.0	0.0
	DF	100.0	0.0	0.0	0.0	0.0	0.0	0.0	0.0	0.0
	JH	0.0	0.0	0.0	2.8	13.9	0.0	0.0	16.7	66.7
	YD	0.0	0.0	0.0	0.0	9.1	0.0	0.0	24.2	66.7
	MN	0.0	0.0	0.0	0.0	0.0	0.0	0.0	0.0	100.0

## Discussion

*Ae. aegypti* is an important vector of dengue fever in China. It was previously thought that this species was only found in China south of latitude 22°N. Climate change, the rapid development of tourism and transportation and increasing urbanization have resulted in an increase in *Ae. aegypti* breeding sites and consequently a marked change in the species’ range in China. In recent years, the range of *Ae. aegypti* has expanded to approximately latitude 25°N [27].

The research presented in this paper is the first study of *kdr* resistance mechanisms in wild *Ae. aegypti* strains in China. We used the bioassay recommended by the WHO to determine the level of pyrethroid resistance of five wild strains of *Ae. aegypti*. The results show that the three wild strains obtained from Ruili in Yunnan (the Jinghan, Yundang and Munao strains) generally had high pyrethroid resistance. Highest and lowest levels of resistance to cypermethrin were 88.82 and 27.51 times, respectively, that of the susceptible strain, and for cyhalothrin, 88.87 and 30.7 times that of the susceptible strain. These high levels of resistance are probably related to extensive pesticide use in the Ruili region. Although *Ae. aegypti* was only first detected breeding in Yunnan in 2006 [28], pyrethroid insecticides have been extensively used since its discovery for mosquito control. The resultant selection pressure appears to have produced high levels of resistance to pyrethroids; our results show that cypermethrin and cyhalothrin are already unsuitable for controlling *Ae. aegypti* in the Ruili region.

*Ae. aegypti* collected from the Qishui and Dongfang were 7.13 and 6.82 times as resistant to cypermethrin, and 6.24 and 4.82 times as resistant to cyhalothrin, as the susceptible strain, which were much lower as compared to those of the three Ruili strains. The relatively low pyrethroid resistance in these *Ae. aegypti* strains may be the result of limited pyrethroid use in this economically-indigent area.

Our results indicate a significant positive correlation between the frequency of the S989 and V1016 mutations and resistance to cypermethrin and cyhalothrin in the five *Ae. aegypti* strains examined. As such, the higher the frequency of these mutations, the higher the level of resistance to cypermethrin and cyhalothrin one would expect. Lin *et al.* [29] found a correlation between the frequency of the V1016G *kdr* mutation and resistance to transfluthrin, d-allethrin, metofluthrin, esbiothrin and prallethrin. Moreover, the S989P mutation often co-exists with V1016G in pyrethroid resistant strains of *Ae. aegypti* [30]. However, V1016G/S989P individuals are no more resistant to pyrethroids than those carrying just one of the mutations [31]. This suggests that S989P does not enhance the resistance conferred by the V1016G mutation. Du *et al.* [31] speculated that S989P could compensate for any reduction in fitness caused by the V1016G mutation. Hirata *et al.* [32] states that S989P alone did not alter deltamethrin sensitivity, but when combined with V1016G, mosquitoes were ten times more sensitive to deltamethrin. Kawada *et al.* [33] states that S989P has always been found with V1016G, but V1016G has been found alone. S989P has never been found alone. From our results, the frequency of occurrence of S989P and V1016G heterozygotes and homozygotes, we speculate that the V1016G occurred before the S989P mutation.

Hu *et al.* [34] previously confirmed by the insertion of the 1534C equivalent mutation into the sodium channel gene of the cockroach that the F1534C mutation confers sodium channel resistance to type I, but not type II, pyrethroids. The F to C mutation drastically reduced sodium channel sensitivity to three type I pyrethroids; permethrin, NRDC 157 (a deltamethrin analogue lacking the a-cyano group) and bioresemthrin, but not to three type II pyrethroids (cypermethrin, deltamethrin and cyhalothrin). We found that the F1534C mutation was negatively correlated with resistance to cypermethrin and cyhalothrin, which suggests that the higher the frequency of this mutation in mosquito strains, the lower their resistance to these two insecticides; thus, the presence of this mutation not only does not enhance resistance to cypermethrin and cyhalothrin but in fact reduces it.

Research on *Ae. aegypti* in Thailand found, regardless of insecticide exposure status, that no homozygous 1016G mutants expressed the homozygous form of the 1534C mutation [35]. Mosquitoes with the homozygous 1534C mutation are more susceptible to deltamethrin than those heterozygous and homozygous for 1534 F [35]. This is a very interesting result which suggests that the F1534C mutation could potentially compensate for any reduction in fitness caused by the S989P and V1016G mutations.

## Conclusions

The methods we established proved to be highly reliable and should aid future studies aimed at further determining the extent of *kdr* mutations worldwide. The frequency of the S989P, V1016G and F1534C mutations were correlated with resistance to cypermethrin and cyhalothrin, and all three mutations were found to be common in *Ae. aegypti* strains throughout Southern China. Our results also provide the first evidence of the S989P, V1016G and F1534C mutations in *Ae. aegypti* in Southern China, thereby increasing the knowledge of these mutations. The V1016G mutation appears to be the most important mutation in *Ae. aegypti* strains in Southern China at the present time, however, there exists the possibility that other mechanisms were also present and acting to confer resistance.
